# Make a Wish – What Are the Wishes for Clinical Psychology and Psychological Treatment?

**DOI:** 10.32872/cpe.7957

**Published:** 2021-12-23

**Authors:** Winfried Rief, Cornelia Weise

**Affiliations:** 1 Division of Clinical Psychology and Psychotherapy, Department of Psychology, Philipps-University of Marburg, Marburg, Germany

It's the end of the year – and we look back to an enormously challenging year. We went through several restrictions in our professional and private lives, we adapted study programs to legal regulations on behaviour during the Pandemic, we missed the direct personal contacts which in the past used to be so essential to find solutions during debates, and we saw things during Zoom conferences that we didn’t want to see. Our society is experiencing a deep and bitter division that is challenging psychology more than it has in a long time. It is quite understandable that people are annoyed, and have lost the motivation to reflect on the current situation.

But still, it's also the time of the year that stands for dreams and wishes. A world without dreams and hopes and wishes is something we would not want to imagine. To exile such a nightmare, we needed an optimistic outlook which can bring us safely through the year 2022. Therefore, we from CPE encouraged the members of our editorial board to take the time to express some of their wishes. And this is what came back: a new vaccination shot, filled with positive ideas, with perspectives and demands to our professional competences, with visions we would like to follow, and lots of optimism. The wishes show the strength of our collaboration to bring clinical psychology and psychological treatments forward for the benefit of the people. But, read yourself:

*In every European country psychological therapies are less available than they should be. A lesson from the English IAPT programme is that politicians will invest more in therapy if we collect and report outcome data in our routine services. Such data shows our value, our focus on patient benefit, and our openness to learning. My New Year wish is that clinical psychologists will once again lead the field in mental health by showing that we as a group embrace outcome monitoring. Other professionals and funding will follow our leadership.* (**David Clark, UK**)

That psychological treatments and all the strategies for care already available in Clinical Psychology reach everyone in need. (**Christina Botella, Spain**)

*Christmas time makes people focus more than usual on other people's needs, joys and concerns. As clinical psychologists, we are sensitive to the needs of our patients and clients, regardless of the season. May this attitude of listening and openness accompany our work, so that we can accompany those who ask for our professional help.* (**Roman Cieślak, Poland**)

A significant portion of research in clinical psychology is unusable because of incomplete or poor reporting. Descriptions of interventions, particularly complex, psychosocial ones, are often sketchy and just reference a manual. My wish is that clinical research is reported more completely, by actually following (not just declaring to have followed) available reporting guidelines. (**Ioana A. Cristea, Italy/USA**)

*Actually, I have a dozen wishes for clinical psychology, some for our patients or clients, others for ourselves. Here's one: that in the bitter dispute between Covid vaccine supporters and opponents, we can provide empathic communication strategies that both increase willingness to vaccinate and diminish the rifts between supporters and opponents. Who, if not us, should provide such helpful interventions to the rest of society?* (**Andreas Maercker, Switzerland**)

I wish that the awareness, at all levels of our society, of the core value of mental health during the COVID crisis does not disappear once it is over. I also want European countries to invest heavily in research (which will then undoubtedly be published in the excellent CPE ;-) and in the implementation of prevention and intervention programmes to improve mental health that are accessible to all people living on European soil. (**Céline Douilliez, Belgium**)

*A more widespread use of clinimetric strategies in psychological assessment.* (**Giovanni A. Fava, USA/Italy**)

Particularly in current times that challenge mental health, I wish all European clinical psychologists loads of resilience, strength, wisdom and self-care. In my own country (Belgium), clinical psychology is facing several important legislative and organisational transitions. I wish that once this phase of uncertainty and burden has passed, clinical psychology will find itself in a renewed and stronger position to the benefit of our clients. (**Dirk Hermans, Belgium**)

*My wish for 2022 is to be dancing with you all at conference parties again!* (**Tania Lincoln, Germany**)

Romanian wish for clinical psychology: Day by day, in many ways, be more and more personalized, high-tech, and evidence-based, for the sake of people's wellbeing! (**Daniel David, Romania**)

*The role of clinical psychology is not evenly distributed in Europe and not even within countries. Given modern information technology it is technically easy to deliver treatments across borders. However, legal and administrative issues make it hard and sometimes even impossible to share treatments and do research. My wish is that we increase collaboration between countries and reduce administrative burden to facilitate spread of evidence-based treatments within Europe.* (**Gerhard Andersson, Sweden**)

May clinical psychology continue to flourish in the year 2022, contribute to an understanding of the basic processes of the development of psychopathology and the principles of change in the treatment of psychological disorders (and help to overcome the pandemic!). (**Bernhard Strauss, Germany**)

*I wish that we develop clear and agreed upon competencies of clinical psychologists that would help our profession and training of the next generation of clinical psychologists.* (**Maria Karekla, Cyprus**)

I would like Santa to become a spiritual member of the CPE team to help us make inter-European networking in the field of clinical psychology even more vibrant, to keep our fire of curiosity burning, and to remind us of the importance of bringing hope and light to those who need us. (**Robert Masten, Slowenia**)

*Wishing happy, healthy and peaceful lives for all. May we feel connected with our hearts and one another, during the Holidays and throughout the new year 2022!* (**Jolanda Meeuwissen, The Netherlands**)

I wish more kindness in this world because we are all part of the same beautiful miracle. Love, peace, and compassion. (**Stefan Hofmann, Germany/USA**)

*2022, please give us healthy clinical psychologists for research and practice in Europe and around the world*. (**Anonymous**)

Personally, I have only the wish for "more time" (we need two more hours per day and an extra free day per week). Professionally, I wish: more collaboration [national, international (European)] in large scale studies; more research on moderators of treatment outcome in different groups of disorders; more research on mediators (mechanism) of change using psychological and biological basic science results; more support and funding for young (female) scientists; more replication studies; less egoism and competition; and again, more personal meetings. (**Martin Hautzinger, Germany**)

*What a time we have all had! I’d like to wish all of you and your families and friends in Europe as well as further afield a restful time over the coming weeks, so that we can embrace 2022 with renewed energy. Carpe diem.* (**Trudie Chalder, UK**)

I wish us all much inspiration in 2022 in generating new ideas to improve the impact of treatments for mental disorders, because that is what people suffering from these conditions very much need. (**Pim Cuijpers, The Netherlands**)

*I wish for Clinical Psychology Research to rapidly develop and empirically validate even better treatments for those with co-morbid chronic physical conditions – and for these to be recognized and implemented by national health care systems.* (**Claus Vögele, Luxembourg**)

I send out a wholehearted thanks to all the psychological therapists who have managed to support and help people in mental health need by delivering treatments online or in person, whilst they themselves have often had challenges in their own lives and at home. My wish for 2022 is that clinical psychology can be at the forefront of preventing mental health problems across Europe and beyond. (**Colette Hirsch, UK**)

*I wish Clinical Psychology in Europe (and beyond) a contagious optimism and resilience in 2022.* (**Omer Van den Bergh, Belgium**)

I wish you all merry Christmas with, hopefully, some face-to-face gatherings with your loved ones. (**Claudi Bockting, President of the EACLIPT**)

Now it’s your turn: take a minute and express your wish for your professional engagement during the year 2022.

Finally to us: For 2022, we wish that our journal will again receive as much support as this year, be it from authors, reviewers, editors, readers, and the excellent team of our publisher PsychOpen, so that we can continue to strengthen the visibility of the many facets of clinical psychology in Europe. As Editors-in-Chief of Clinical Psychology in Europe, we promise to address all your and our wishes to the corresponding institution (see photo).

We wish you a Happy Holiday season and a peaceful and prosperous New Year.

**Figure:**
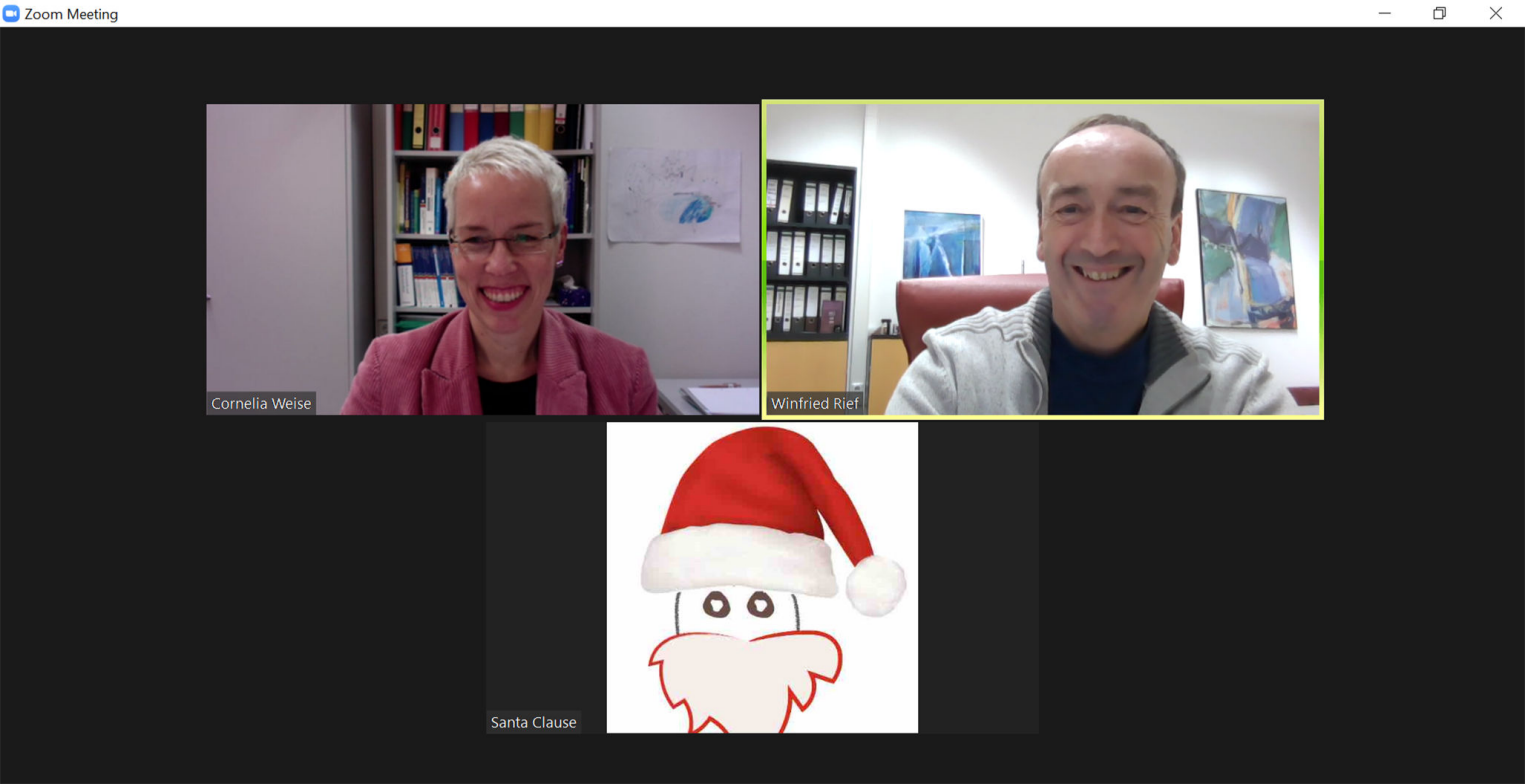




**Winfried Rief & Cornelia Weise**



